# Chronic Effects of Effective Oral Cannabidiol Delivery on 24-h Ambulatory Blood Pressure and Vascular Outcomes in Treated and Untreated Hypertension (HYPER-H21-4): Study Protocol for a Randomized, Placebo-Controlled, and Crossover Study

**DOI:** 10.3390/jpm12071037

**Published:** 2022-06-24

**Authors:** Marko Kumric, Josko Bozic, Goran Dujic, Josip Vrdoljak, Zeljko Dujic

**Affiliations:** 1 Department of Pathophysiology, University of Split School of Medicine, 21000 Split, Croatia; marko.kumric@mefst.hr (M.K.); josko.bozic@mefst.hr (J.B.); josip.vrdoljak@mefst.hr (J.V.); 2 Clinical Department of Diagnostic and Interventional Radiology, University Hospital of Split, 21000 Split, Croatia; goran.dujic@gmail.com; 3 Department of Integrative Physiology, University of Split School of Medicine, 21000 Split, Croatia

**Keywords:** hypertension, cannabidiols, heart rate variability, arterial stiffness, DehydraTECH2.0

## Abstract

Accumulating data from both human and animal studies suggest that cannabidiol (CBD) may be associated with improved cardiovascular function, markedly with regard to reduction in blood pressure and improved endothelial function. However, there is a lack of randomized studies to support these notions, especially in at-risk populations. The principal aim of this randomized, placebo-controlled, and crossover study is to examine the influence of chronic CBD administration on 24-h blood pressure in individuals with mild or moderate hypertension who are either untreated or receiving standard care therapy. The secondary aims of the study are to determine the safety and tolerability of 5 weeks of CBD administration, and to quantify the effect on arterial stiffness, CBD and vascular health biomarkers, inflammation, heart rate variability, and psychological well-being in both groups of patients. The present single-center study is designed as a triple blind (Participant, Investigator, Outcomes Assessor), placebo-controlled, crossover pilot study in which 70 hypertensive volunteers (aged 40–70 years) will receive DehydraTECH2.0 CBD formulation and placebo in a crossover manner. We believe that comprehensive analyses that will be performed in the present trial will decipher whether CBD is in fact a safe and valuable supplement for patients with treated and untreated hypertension.

## 1. Introduction

Cannabidiol (CBD) is now known to serve as an anti-anxiety agent in both animal and human models of stress [1,2]. Stressful situations are associated with increases in blood pressure and heart rate, whereas the meta-analysis by Sultan et al. revealed that CBD can attenuate both [3]. In a recent study, conducted in otherwise young and heathy volunteers, daily CBD (600 mg, p.o.) after both acute and repeated treatment (for seven days) reduced systolic blood pressure (SBP) [4]. Nevertheless, the SBP reduction was only evident during isometric handgrip stress exercise and not at rest [4]. In the same study, it was demonstrated that repeated CBD dosing decreased arterial stiffness and improved endothelial function [4]. It would seem reasonable, however, that the potential hypotensive effect of CBD may be better observed in individuals during the early development of hypertension, a common condition that is normally reflective of arterial stiffness and a compromise in endothelial function [5].

The influence of CBD on the cardiovascular system in humans might depend not only on a dose and duration of administration, but also on the delivery method of CBD [4]. For example, oral CBD at dose of 90 mg did not influence BP, heart rate (HR), and cerebral perfusion, but the same dose of CBD encapsulated as TurboCBD^TM^ (patented capsule formulation increasing CBD bioavailability) decreased diastolic BP (DBP) and mean BP (MBP) and increased cerebral perfusion [6]. TurboCBD^TM^ formulation included 45 mg or 90 mg CBD, in 120 mg or 240 mg of hemp oil, together with 600 mg or 1200 mg American ginseng and 240 mg or 480 mg ginkgo biloba, respectively. Most recently, in volunteers with pre-hypertension, we observed significant reductions in SBP and MBP, as well as arterial stiffness, over an ambulatory 24-h monitoring period following 150 mg of oral CBD DehydraTECH2.0 administered on a single day every 6–8 h (totalling 450 mg) when compared to placebo (manuscript in preparation). This study used a DehydraTECH2.0 CBD formulation designed to increase CBD bioavailability, especially with regard to delivery into brain tissues, which could in turn enable greater effectiveness upon neurogenic hypertension mechanisms operating in the early stages of hypertension. However, the impact of more chronic CBD administration over a period of weeks on 24-h blood pressure, vascular health, autonomic nervous system regulation, and biomarkers of oxidation and inflammation has not been examined in patients with early, untreated hypertension. Moreover, it is unknown if CBD may further improve these measures in patients already on standard care blood pressure medication who commonly remain mildly hypertensive. This lack of information is surprising given the involvement of the cannabinoid system in several aspects of cardiovascular regulation including control of blood vessel tone, cardiac contractility, blood pressure, and vascular inflammation [7,8,9].

The principal aim of this randomized, placebo-controlled, and crossover study is to examine the influence of chronic CBD administration on 24-h blood pressure in individuals with mild or moderate hypertension who are either untreated or receiving standard care therapy. The hypothesis is that the hypotensive effects of CBD will be apparent in both untreated and treated hypertension. The secondary aims of the study are to determine the safety and tolerability of five weeks of CBD administration, and to quantify the effect on arterial stiffness, CBD and vascular health biomarkers, inflammation, heart rate variability, and psychological well-being in both groups of patients.

## 2. Materials and Methods

### 2.1. Study Design and Setting

**Figure 1:**
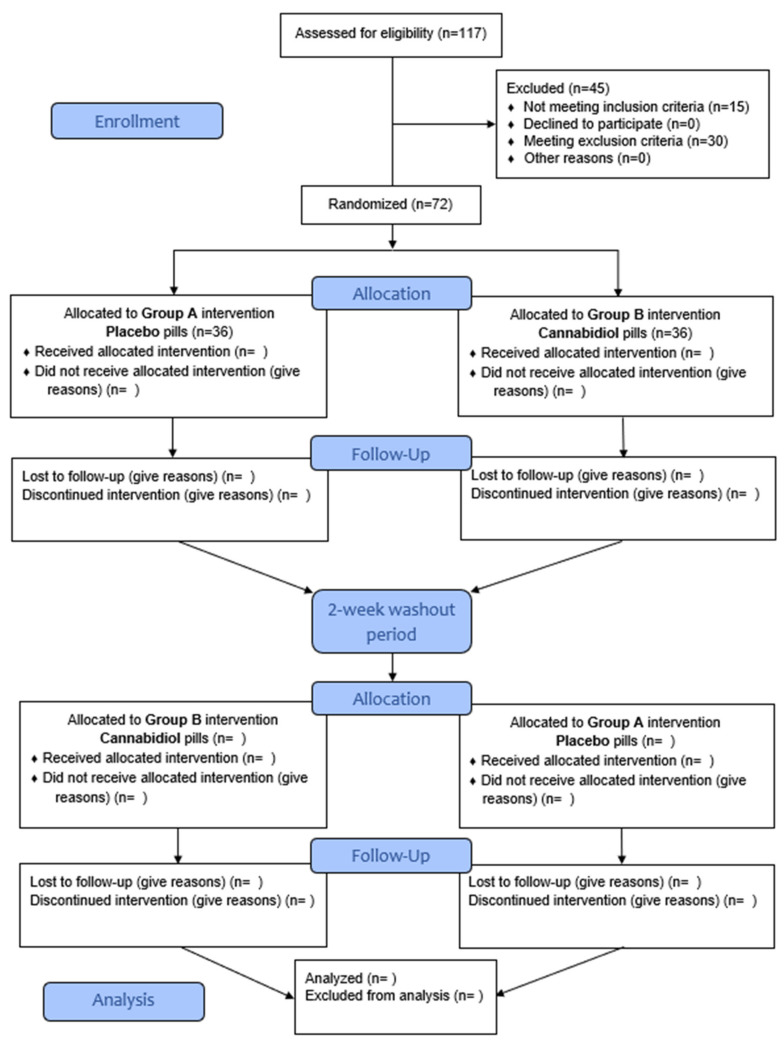
CONSORT flow diagram.

The present single-center study is designed as a triple blind (Participant, Investigator, Outcomes Assessor), placebo-controlled, crossover pilot study in which 70 hypertensive volunteers (aged 40–70 years) will receive DehydraTECH2.0 CBD formulation and placebo in a cross-over manner. The study protocol was registered at the ClinicalTrials.gov (accessed on 7 April 2022) (NCT05346562). The sequence of conditions the participant will receive will be generated by a research randomizer web-service (https://www.randomizer.org, accessed on 7 April 2022) and participants will complete: (1) placebo-control and (2) DehydraTECH^TM^ CBD (225 to 300 mg split over three times daily for the initial 2.5 weeks and 375 to 450 mg split over three times for the following 2.5 weeks). Following a two-week washout, participants will then repeat testing for another five weeks under different conditions. Participants will visit the laboratory on 6 occasions in total (3 in each study arm), each time following an overnight fast. The complete course of the study will be performed at the Department for Integrative Physiology, University of Split School of Medicine, Split, Croatia. The study design, implementation, and reporting will follow the CONSORT guidelines, including the 25-item checklist and a flow diagram (Figure 1). The study will be conducted according to the guidelines of the Declaration of Helsinki and was approved by the Ethics Committee of University of Split School of Medicine on 15 December 2021 (Class: 003-08/21-03/0003; Reg. No.: 2181-198-03-04-21-0091). Prior to enrollment in the study, every participant was informed about the procedures, course, and purpose of the research and each of them individually signed an informed written consent. 

### 2.2. Eligibility Criteria

#### 2.2.1. Inclusion Criteria

- Documented or measured elevated blood pressure (130/80 to 139/80 mmHg), mild (stage 1) hypertension (140/90 to 159/99 mmHg), or moderate (stage 2) hypertension (160/100 to 179/109 mmHg)
  o At least 30 antihypertensive drug-naïve subjects
  o At least 30 subjects treated with (1) angiotensin converting enzyme (ACE) inhibitors with or without diuretics or (2) ACE inhibitors with calcium channel blocker with or without diuretics
- Normal or overweight (body mass index 18.5 to <30.0 kg/m^2^)
- Between the ages of 40–70 years of age
- Post-menopausal women (at least 24 consecutive months in the absence of medications known to induce amenorrhea)
- Undergoing <150 min of moderate-to-vigorous activity per week
- Heterosexually active female subjects of reproductive potential must be on contraception management using at least one of the following methods while taking study drug and for 30 days following the last dose of study drug:
  o Barrier method of contraception [condoms (male or female), diaphragm, or cervical cap with spermicide
  o Intrauterine device (IUD)
  o Hormone-based oral, injectable, or implantable contraceptive
  o Bilateral tubal ligation/cauterization
  o Surgically sterile (hysterectomy or bilateral oophorectomy) or vasectomized male partner

#### 2.2.2. Exclusion Criteria

- Current smoker or using vapor-based products; or a medical or recreational cannabis user
- Secondary hypertension
- Active malignant disease
- Gout
- History of psychosis and/or depression and/or clinically diagnosed anxiety
- Chronic kidney disease
- Chronic gastrointestinal disease (e.g., irritable bowel syndrome, celiac disease, inflammatory bowel disease, chronic diarrhea) or has had a cholecystectomy in the past
- Current diagnosis or history of any seizure disorder
- Heart failure
- Diabetes mellitus
- Pregnant, breast feeding, or plan to become pregnant
- History of opioid use
- Dual blood pressure therapy other than ACE inhibitors with diuretic or ACE inhibitors with Calcium Channel blocker with or without diuretics (e.g., ACE inhibitors with beta blockers)
- Unwilling or unable to execute the informed consent documentation
- Liver disease, including taking valproate, confirmed on baseline blood biochemistry—exclusion of subjects that have >1.5 upper limit of normal (ULN) for alanine transaminase (ALT) or aspartate transaminase (AST), total bilirubin (TBL) > ULN at baseline determined by local reference population values in Croatia:
  o ULN for ALT 48 IU/L
  o ULN for AST 38 IU/L
  o ULN for TBL 20 μmol/L

The above noted inclusion/exclusion criteria are assessed with a medical screening questionnaire during a clinical examination and related baseline blood chemistry. If at any point during the trial participants meet any of the following criteria, they will be excluded:

- ALT or AST > 3x ULN accompanied by fever, rash, fatigue, nausea, vomiting, right upper quadrant pain or tenderness, and/or eosinophilia (>5%)
- ALT or AST > 5x ULN
- ALT or AST > 3x ULN and TBL > 2x baseline

### 2.3. Study Arms and Interventions 

Participants allocated in the first arm (Cannabidiol, then Placebo) will first receive cannabidiol: 225 to 300 mg (depending on the sex and weight of the participants) split over three times daily for the initial 2.5 weeks and 375 to 450 mg split over three times for the following 2.5 weeks. After two-week washout, participants will receive Cannabidiol-matched placebo tablets for five weeks. Participants allocated in the second arm (Placebo, then Cannabidiol) will receive Cannabidiol-matched placebo tablets for five weeks. After two-week washout, participants will receive cannabidiol: 225 to 300 mg split over three times daily for the initial 2.5 weeks and 375 to 450 mg split over three times for the following 2.5 weeks. A detailed dosing regime is presented in Table 1. The production of both placebo and capsules containing CBD was done in accordance with the standard operating procedures by experienced chemical engineers. The manufacturing process was performed using the services of a Good Manufacturing Practices (GMP)-compliant contract manufacturer located in the United States. The DehydraTECH2.0 CBD formulation was comprised of Lexaria Bioscience Corp.’s patented mixture of long chain fatty acid rich triglyceride oil and tetrahydrocannabinol-free, purified cannabidiol distillate oil, together with organic substrate powder ingredients and other organic constituents included to enhance intestinal absorption performance. The DehydraTECH2.0 CBD formulation was prepared as a powder and filled into Size 00 vegan gel capsules at a target strength per capsule of 75 mg CBD as independently verified via high-performance liquid chromatography (HPLC) upon quality control testing. The DehydraTECH2.0 CBD formulation used in the present study was also subjected to Lexaria Bioscience Corp.’s patented performance-enhancing dehydration processing methodology. The placebo capsules were simply filled with an organic substrate powder ingredient devoid of any CBD active drug substance as also verified upon HPLC testing, and similarly filled into matching Size 00 vegan gel capsules for blinding purposes. 

Each subject will be provided with a diary to confirm the timing of each daily dose of the CBD or placebo capsules. Additionally, during each visit, physical activity and food intake diaries (24 h) will be provided for each participant. For experimental visits 2–6, participants will be instructed to follow the same pattern of both physical activity and food intake as during visit No. 1. In the event of a missed dose, a maximum of one capsule (75 mg) will be added to the next dose. Apart from insight in the dosing diary, adherence will be assessed by analyzing the blood and urine CBD and its metabolites.

### 2.4. Outcome Measures 

The primary outcome is 24-h ambulatory blood pressure measured by ambulatory blood pressure monitoring system Schiller BR-102 plus PWA (Schiller AG, Baar, Switzerland). The device will be set to take measurements every 30 min through the day (8 a.m.–11 p.m.) and every hour through the night (11 p.m.–8 a.m.) [10]. Participants will be asked to keep their arm still at the level of their heart when the device starts to take a reading.

The secondary outcomes are:

1. Cannabidiol levels in peripheral blood sample.
2. Safety (via blood biomarkers, #8 and tolerability (via adverse event reporting).
3. Calories burned, total sleep time, number of awakenings—Physical activity and sleep quality (actigraphy) will be monitored via a wrist-based health and fitness tracker FitBit (Fitbit Inc., San Francisco, CA, USA) with a large organic light-emitting diode screen featuring heartrate monitoring and tracking of steps, distance, calories burned, floors climbed, active minutes, and sleep duration. These devices have been well-validated against gold-standard measures with good accuracy [11].
4. Pulse wave velocity, augmentation index, and total peripheral resistance measured by Schiller BR-102 plus PWA (Schiller AG, Baar, Switzerland).
5. Volume of hippocampus and internal carotid artery blood flow—Volume of hippocampus will be assessed by magnetic resonance imaging (MRI), T1-MPRAGE sequence, whereas blood flow through internal carotid artery will be measured by 1.5T MRI, 4D flow.
6. Heart rate variability measured by Schiller medilog^®^AR Holter recorder (Schiller AG, Baar, Switzerland).
7. Cardiovascular risk assessed by tissue levels of advanced glycation end-products (AGEs)—The AGE Reader (DiagnOptics Technologies BV, Groningen, The Netherlands) is a non-invasive monitoring device that uses ultra-violet light to excite autofluorescence in human skin tissue. The autofluorescence reflects tissue levels of AGEs [12]. The measurement of AGEs provides an immediate cardiovascular risk prediction in 12 s. The AGE Reader has been designed for patient-friendly diagnosis; the method is convenient, easy to use, and extensively validated.
8. Circulating levels of blood biomarkers—Circulating (steady-state) plasma concentrations of CBD, inflammation (including TNF-alpha, IL-6, IL-10, whole blood count, etc.) and gold-standard biomarkers of plasma nitric oxide (plasma nitrite, s-nitrosothiols, and total nitric oxide) will be measured from peripheral blood sample. Lipids (cholesterol, high- and low-density lipoprotein, and triglycerides), glucose, insulin, HbA1C, renal (creatinine), hepatic (ALT, AST, ALP, GGT, and total bilirubin), drug toxicity (acyl glucuronide), and cardiovascular disease markers (hs-CRP) will also be measured.
9. Outcomes assessed using questionnaires:
  - Salt intake measured by Big life sodium calculator [13]
  - Sleepiness measured by Epworth sleepiness scale [14]
  - Geriatric depression measured by Geriatric depression scale-short form [15]
  - Physical activity measured by Global Physical Activity Questionnaire (Total weekly Metabolic equivalent of task (MET)-min is calculated in a following manner: (Minutes engaged in moderate-intensity activity each week X 4 MET) + (Minutes engaged in vigorous-intensity activity each week X 8 MET) [16]
  - Memory measured by Memory Complaint Questionnaire [17]
  - Sleep quality measured by Pittsburgh sleep quality index [18]
  - Perceived stress measured by Perceived stress scale [19]
  - General health measured by Short form-36 (SF-36) [20]
  - Anxiety measured by State-Trait Anxiety Inventory [21]
  - Obstructive sleep apnea risk measured by STOP-BANG [22]
  - Depression measured by Beck’s Depression Inventory [23]
  - Mediterranean diet serving score [24]

### 2.5. Laboratory Visits

The initial screening visit was comprised of initial assessment of anthropometrics (height, weight, waist and neck circumference), blood pressure and relevant vital signs, assessment of eligibility for study participation, and obtaining the past medical history. A total of 117 volunteers were screened, 15 patients did not meet the inclusion criteria and 30 patients met the exclusion criteria. After the completion of the screening procedures, eligible participants were randomly assigned to one of the two experimental groups. Following randomization, participants will undergo their first set of physiological, neuroimaging, sleep, and cognitive assessments. Additionally, participants will complete several lifestyle and psychological questionnaires (Table 2). This battery of assessments will be completed again following 2.5 weeks and after 5 weeks, and then repeated immediately after the washout period, 2.5 weeks after that, and 5 weeks after finishing the washout period. There will be 6 experimental visits in total to the laboratory, with additional 4 visits to the MRI suite (in selected subjects that will undergo neuroimaging). Participants will report to the laboratory after an overnight fast (>10 h) and around 07:30. Of note, subjects will be reminded to not perform exercise or to consume alcohol 24 h prior to the next visit. Water intake will be measured and allowed *ad lib*. During each experimental visit, a sample of venous blood will be taken from the forearm (cubital vein) using a sterile disposable needle and a previously cleansed skin surface. All blood samples will be procured in the early morning visit, prior to consuming the CBD or placebo capsules. This procedure will be performed by a trained laboratory technician. A maximum of 3 to 22 mL of blood will be drawn. Part of the sampled blood will be sent for immediate analysis, whereas part of the sample will be aliquoted and stored on −80 °C for subsequent analysis of biomarkers. All blood samples will be analyzed in the same certified institutional biochemical laboratory, by using the standard operating procedures. In addition, a sample of urine will be collected and stored for analysis of CBD and its metabolites with LCMS in plasma and urine.

Following the extraction of the initial blood sample, subjects will be evaluated using bioelectrical impedance scale Tanita DC-360 S (Tanita, Tokyo, Japan) and AGE Reader and then instrumented with the 24-h ambulatory blood pressure monitor that will be used to assess continuous blood pressure measurements away from the laboratory. The device will be set to take measurements every 30 min through the day (08:30–23:00) and every hour through the night (23:00–8:00). Subjects will be also instrumented with 24-h ECG monitor. Subjects will also be asked to wear a Fitbit activity and sleep monitor. Finally, prior to discharge, subjects will be given an appropriate set of questionnaires. Apart from the bioelectrical impedance analysis which will be performed only during experimental visit 1 and 4, and AGE Reader measurement, which will be performed during visits 1, 3, 4, and 6, the protocol will be the same for all experimental visits. The whole procedure will take about 40 min per visit.

### 2.6. Sample Size Calculation

Sample size analysis has been conducted using data from our previously published pilot study in which similar active intervention and outcomes were investigated [6]. The calculated required sample size for the present analysis was 60 patients. Nevertheless, this study will recruit a total of 72 participants, which will include a split of those who are either untreated or receiving guideline-recommended antihypertensive therapy. This power calculation, which is based on a sample size calculation formula for a mixed model for repeated measures data with attrition, provides 80% power to detect 0.4 standard deviation between group difference for an average repeated measurement correlation of 0.6 and three repeated measurements, taking up to 20% attrition into consideration [24].

### 2.7. Adverse Events

The present study confers a minimal risk of adverse events (AE). The safety (lack of AEs) of acute dosing of 300 mg DehydraTECH2.0 CBD has been established; and previous studies have used much higher acute dosing without reports of related AEs [4]. Specifically, the final daily dose (450 mg, approx. equally to 4.5 mg/kg/day) is 2–4-fold less than the reported 1500 mg/kg/day of CBD (or 10–20 mg/kg/day) used in the recent Phase 1 trial by Watkins et al. that revealed peak serum alanine aminotransferase values were above the upper limit of normal in 7 (44%) out of the 16 participants and exceeded international criteria for drug-induced liver injury in 5 (31%) of these participants [25]. In the aforementioned study, the most common all-causality AEs by preferred term were gastrointestinal disorders, including diarrhea in eight (50%) participants and abdominal discomfort in five (31%) participants. Most of these AEs were first experienced during the CBD titration phase. All reported AEs were either mild (31%) or moderate (50%) in severity. Of note, CBD is a commercially available dietary supplement. Nevertheless, we will monitor both AEs and serious AEs (SAEs). Subjects are instructed to contact a study team member if any AE occurs and permission to reach out to emergency contacts has been requested from all participants in case of the subjects not being responsive. The principal investigator of the study, alongside two study investigators holding a medical degree, will continuously monitor the conduct, data, and safety of the study, including monitoring and tabulating AEs. Potential AEs will be evaluated in accordance with the Common Terminology Criteria for Adverse Events (CTCAE) V.5.0 and AEs will be graded based on the likelihood of being related to the study intervention [26]. MedDRA PT (version 24.1) will be used for documenting any AE and serious AEs. All SAEs will be reported to the local Institutional Review Board (IRB) according to good clinical practices within 24 h for potentially life-threatening events and within 5 business days for non-life-threatening events. If such event occurs, the participant will immediately stop receiving treatment and be excluded from study. Additionally, an internal safety monitor will adjudicate all SAEs for report completeness, seriousness of event, and relationship to study interventions. The trial will terminate immediately if 20% of involved participants report SAEs.

### 2.8. Data Collection and Management

Obtained data of interest will be recorded during each visit and entered on a special research form (respondents’ card) by study researchers, and it will subsequently be archived and protected by a unique code of the participant. For keeping data, we will use a paired digital archive consisting of computer’s internal memory and external hard drive. The lab book will be kept at the Department of Integrative Physiology, and after the research is completed, the raw data will be available on request from the corresponding author. The data will not be publicly available because some of the dataset will be used for further research.

### 2.9. Statistical Analysis Plan

Collected data will be analyzed with the statistical software SPSS statistics (version 21.0, IBM, Chicago, IL, USA) and Prism 6 for Windows^®^ (version 6.01, GraphPad, La Jolla, CA, USA). Quantitative data will be expressed as mean ± standard deviation or median and interquartile range, as appropriate, while qualitative data will be expressed as a whole number and percentage. Kolmogorov–Smirnov test will be used to estimate the normality of data distribution. A comparison of quantitative variables will be conducted using Student’s *t*-test for independent samples, or Mann–Whitney U tests where appropriate. Data will be analyzed for repeated measures analysis using either general or mixed linear models (with sex, body mass, medication, and age as a co-variates). Yet, the final analysis plan, which includes analyses of the secondary outcomes and strategies for handling missing/incomplete data, will be formalized by the biostatistician prior to breaking the blind. The data analysis will only begin once data collection is complete. Significance will be set at *p* < 0.05 for all comparisons.

## 3. Rationale for Conducting the Trial

Accumulating data from both human and animal studies suggests that CBD may be associated with improved cardiovascular function, markedly with regards to reduction in blood pressure and improved endothelial function [27,28,29]. Nevertheless, there is a lack of randomized studies to support these notions, especially in at-risk populations. Therefore, in the present trial, the primary endpoint will be reduction in 24-h ambulatory blood pressure. Specifically, we will assess its effects on both treated and treatment-naïve patients with essential hypertension. In order to further elucidate and quantify the effects of CBD, we will explore its effects on arterial stiffness, serum levels of endothelial dysfunction biomarkers, and autonomic nervous system modulation on the cardiac sinus node. In addition, using the panel of validated questionnaires, the psychological well-being will be determined in both groups of patients. We believe that comprehensive analyses that will be performed in the present trial will decipher whether CBD is in fact a safe and valuable supplement for patients with treated and untreated hypertension. In addition, understanding new approaches to CBD delivery has major implications for more precise recommendations of CBD usage in health and disease states.

## Figures and Tables

**Table 1:** Dosing schedule.

Randomization Group	Dose Period 1 (2.5 Weeks)	Dose Period 2 (2.5 Weeks)
DehydraTECH2.0 CBD	***≤75 kg: CBD, 225 mg/day***	***<100 kg: CBD, 375 mg/day***
	75 mg morning (1 capsule)	75 mg morning (1 capsule)
	75 mg afternoon (1 capsule)	150 mg afternoon (2 capsules)
	75 mg bedtime (1 capsule)	150 mg bedtime (2 capsules)
	***>75 kg: CBD, 300 mg/day***	***≥100 kg: CBD, 450 mg/day***
	75 mg morning (1 capsule)	150 mg morning (2 capsules)
	75 mg afternoon (1 capsule)	150 mg afternoon (2 capsules)
	150 mg bedtime (2 capsules)	150 mg bedtime (2 capsules)
Placebo	Placebo, number of capsules matched to active treatment based on body weight	Placebo, number of capsules matched to active treatment based on body weight

Abbreviations: CBD: cannabidiol.

**Table 2:** Schedule of measurements.

Assessment Measure	Screening	First Set of Visits			Washout	Second Set of Visits		
Timing of visit (weeks)		0	2.5	5		0	2.5	5
**Anthropometry and medical history**								
Medical history	✓							
Demographics and health history	✓							
Eligibility screening questionnaire	✓							
Anthropometrics and vital signs	✓	✓	✓	✓		✓	✓	✓
Bioelectrical impedance analysis		✓				✓		
**Indices of cardiovascular health**								
24-h ambulatory blood pressure		✓	✓	✓		✓	✓	✓
24-h ambulatory ECG		✓	✓	✓		✓	✓	✓
Pulse wave analysis		✓	✓	✓		✓	✓	✓
AGE Reader		✓						
Brain structure and function (MRI)		✓		✓		✓		✓
**Blood work and urine analysis**								
Blood biomarkers		✓		✓		✓		✓
Liver transaminases	✓	✓	✓	✓		✓	✓	✓
Cannabidiol in urine		✓	✓	✓		✓	✓	✓
**Sleep, health and physical activity**								
Actigraphy		✓	✓	✓		✓	✓	✓
Sleep quality		✓	✓	✓		✓	✓	✓
**Questionnaires**								
Big life sodium calculator		✓		✓		✓		✓
Epworth Sleepiness Scale		✓		✓		✓		✓
Geriatric Depression Scale		✓	✓	✓		✓	✓	✓
Global Physical Activity Questionnaire		✓						
Memory Assessment Clinic-Q		✓				✓		
Mediterranean Diet Serving Score		✓						
Pittsburgh Sleep Quality Index		✓						
Perceived Stress Scale		✓		✓		✓		✓
Short Form-36		✓		✓		✓		✓
State-Trait Anxiety Inventory		✓		✓		✓		✓
STOP-Bang		✓	✓	✓		✓	✓	✓
Beck’s Depression Inventory		✓						

Abbreviations: ECG: electrocardiogram; MRI: magnetic resonance imaging; AGE: advanced glycation end-products.

## Data Availability

The data presented in this study are available on request from the corresponding author. The data are not publicly available because some of the dataset will be used for further research.
